# Extra Virgin Olive Oil: Lesson from Nutrigenomics

**DOI:** 10.3390/nu11092085

**Published:** 2019-09-04

**Authors:** Stefania De Santis, Marica Cariello, Elena Piccinin, Carlo Sabbà, Antonio Moschetta

**Affiliations:** 1 Department of Interdisciplinary Medicine, University of Bari “Aldo Moro”, 70124 Bari, Italy; 2 INBB, National Institute for Biostructures and Biosystems, 00136 Rome, Italy; 3 Department of Pharmacy-Drug Science, University of Bari “Aldo Moro”, 70126 Bari, Italy; 4 IRCCS Istituto Tumori Giovanni Paolo II, 70124 Bari, Italy

**Keywords:** extra virgin olive oil, polyphenol content, fatty acids, nutrigenomics, human health

## Abstract

Extra virgin olive oil (EVOO) consumption has a beneficial effect on human health, especially for prevention of cardiovascular disease and metabolic disorders. Here we underscore the peculiar importance of specific cultivars used for EVOO production since biodiversity among cultivars in terms of fatty acids and polyphenols content could differently impact on the metabolic homeostasis. In this respect, the nutrigenomic approach could be very useful to fully dissect the pathways modulated by different EVOO cultivars in terms of mRNA and microRNA transcriptome. The identification of genes and miRNAs modulated by specific EVOO cultivars could also help to discover novel nutritional biomarkers for prevention and/or prognosis of human disease. Thus, the nutrigenomic approach depicts a novel scenario to investigate if a specific EVOO cultivar could have a positive effect on human health by preventing the onset of cardiovascular disease and/or chronic inflammatory disorders also leading to cancer.

## 1. Introduction

Extra-virgin olive oil (EVOO) is an essential food of the Mediterranean diet (MD) and countries in the Mediterranean area like Spain, Italy, and Greece represent the most important producers worldwide [1]. MD is characterized by a nutritional model consisting mainly of a high consumption of EVOO, vegetables, fruits, cereals, nuts, and legumes, a moderate intake of proteins from fish, meat, and dairy products along with red wine, and a low intake of eggs and sweets [2]. Moreover, differently from other dietary treatments, MD represents a net of traditions and knowledge and for this reason it could be considered a way of life rather than just a food. For all these reasons, in 2013 the UNESCO inscribed MD on the representative list of the Intangible Cultural Heritage of Humanity (https://ich.unesco.org/en/RL/mediterranean-diet-00884). Moreover, adherence to MD is associated with longevity and a lower incidence of chronic diseases [3].

As documented by numerous studies published over the past decades, most of the beneficial effects of MD on human health promotion can be ascribed to EVOO. In fact, consumption of olive oil is able to reduce lipid and DNA oxidation, ameliorate lipid profile and insulin-resistance, endothelial dysfunction, inflammation, and to lower blood pressure in hypertensive patients. These effects protect from both cardiovascular disease and metabolic disorders [4,5]. For this reason, we decided to study the state-of-art of EVOO focusing on the differences existing among cultivars that can have a different impact on the human health mainly through the modulation of human transcriptome.

## 2. EVOO Cultivars and Biodiversity: Effect on Health Promotion

Traditionally, the beneficial properties of EVOO have been attributed to its high monounsaturated fatty acid (MUFA) content, that account for up to 80% of its total lipid composition. However, recently cumulative evidences have shown that the minor components of EVOO, as phenolic compounds and other compounds with antioxidant characteristics, may also contribute to the healthy features of EVOO [6]. These components make up only 1–2% of EVOO, but they are completely absent in other type of oils derived from seeds or fruits [7].

The quality and the organoleptic properties of EVOO depend on different factors such as cultivar, geographic origin, climatic conditions, agronomic and processing techniques that are able to modify fatty acids composition and bioactive compounds concentration. More than 5500 years ago, the variety of olive tree *Olea europea* L. was probably one of the first domesticated trees that followed the migration routes of the populations in the Mediterranean area [8]. Today, people cultivate olives thanks to healthy centenary or millenary huge sized trees. For example, cultivar *Pisciottana* has been introduced in the area of Cilento and Vallo di Diano (SA, Italy) 2000 years ago by Phocaeans. The olive trees have an enormous phenotypic and genetic variability [9]. By now, in the world, it is possible to distinguish more than 600 cultivars excluding synonymies and homonymies [10]. To protect the EVOO according to its origin, genetic, and phenotypic characteristics, the European Union provides the Protected Designation of Origin (PDO), Protected Geographical Indication (PGI), and traditional specialties guaranteed (TSG), important information contained in the label [11]. PDO is granted to morphologically and genetically distinct oils with a regional specificity that gives a peculiar quality to the product [12]. Although in the past decades the genetic characterization of several olive varieties has been carried out with different molecular tools, other studies are still needed to fully typify the peculiarities related to a specific cultivar [13]. 

It has been demonstrated that fatty acids composition of the EVOO is mainly genotype-dependent [14]. In general, Greek, Italian, and Spanish EVOO are low in linoleic and palmitic acids and rich in oleic acid [15]. *Leccino*/*Frantoiana*, *Salella*, and *Pisciottana* cultivars displayed a peculiar FA profile characterized by high levels of behenic, linoleic, and heptadecenoic acids, respectively [16]. In particular, the *Salella* variety is characterized by a high concentration of omega-6 fatty acids promoting the reduction of total blood cholesterol and low density lipoprotein (LDL) levels and supporting human health [10]. Ghisoni et al. through liquid chromatography and mass spectrometry metabolomics underlined the importance of geographical origin by analyzing cultivars from Sicily, Apulia, Umbria, Liguria, and Tuscany grown in the same conditions. The authors observed that several sterols derivatives (stigmasterol and furostanols) are featured in the cultivars considered [17]. Furthermore, a recent study, evaluated the effects of chylomicron remnant-like particle (CRLP) enriched in fatty acids of EVOO obtained from *Chetoui*, *Buldiego*, *Galega*, *Blanqueta*, and *Picual* cultivars on the foam cells formation via THP-1 macrophages [18]. *Chetoui* and *Blanqueta* cultivars (riches in linoleic acid) induced higher total triacylglycerol (TAG) incorporation into THP-1 cells than *Buldiego* and *Picual* (riches in oleic acid) promoting foam cells formation [18]. This parameter can be used to identify EVOO with possible cardioprotective effects. 

Nutritional and anti-oxidant properties of EVOO are related to the presence and concentration of tocopherols, carotenoids, and phenolic compounds which are of great importance on human health [19]. Notably, the Mediterranean countries presented the lowest intake of total polyphenols compared to non-Mediterranean countries and the United Kingdom [20]. Moreover, polyphenols in Mediterranean countries derive from coffee, fruits, wine, and vegetables oils, whereas in the non-Mediterranean countries, these compounds come from coffee, tea, and wine [20]. Olive oils contain different classes of phenolic compounds such as phenyl ethyl alcohol (hydroxytyrosol and tyrosol), cinnamic (caffeic acid and p-coumaric acid) and benzoic (vanillic acid) acids, flavones (apigenin and luteolin), and secoiridoids (oleuropein and ligtroside derivatives) [21,22,23]. Several works showed that the beneficial properties of EVOO are due to the phenolic component [24] that confers to the EVOO free radical scavenging activity. The main polyphenol in EVOO, hydroxytyrosol, is a ROS scavenger that reduces oxidized LDL and platelets aggregations [25]. Oleuropein is an anti-inflammatory molecule that promotes nitric oxide production in macrophages [26]. Oleocanthal exerts anti-inflammatory properties similar to ibuprofen [27]. Polyphenols-rich EVOO is able to reduce heterocyclic amines and plasmatic C-reactive protein levels [28,29], and ameliorate lipid metabolism and platelets function [30,31] as well as glycaemia and insulin sensitivity [32]. Most of the effects of the phenolic compounds are due to their in vivo bioavailability [33]. Indeed, during the digestive process these molecules are lost between buccal and duodenal tracts. However, differences in the phenolic bioavailability have been observed in different cultivars of virgin olive oil (VOO), thus suggesting that the variety is essential to determine the biological and beneficial properties of the oils [34]. A very recent study evaluated the effects of phenolic compound of *Tonda di Cagliari* cultivar in the protection of Caco-2 cells from oxidative stress demonstrating that hydroxytyrosol, oleuropein, and verbascoside inhibited ROS generation and reduced membrane oxidative damage [35]. A pilot study on healthy subjects showed that administration of 12 g of table olives *Nocellara del Belice* variety modulated systemic inflammation reducing IL-6 and MDA (poly-unsaturated fatty acids peroxidation product) levels [36].

Notably, the chemical composition of EVOO is affected by environmental conditions, agronomic practices, harvesting handlings, and extraction systems [37]. The type of extraction procedure of the oil is the process that mostly influences the concentration of phenolic compounds. The EVOO is obtained by manually crushing and processing olives, thus preserving the content of minor components that are irreversibly lost in refined olive oil, extracted with both chemical and physical procedures. Notably, despite the similar amount of MUFA displayed by both oils, when compared to EVOO, refined olive oil shows less healthy effect, because of the low phenolic content [38]. Ambra et al. studied tocopherols, β-carotene, lutein, squalene, and polyphenols in Italian EVOO monocultivars (*Leccino*, *Rustica*, *Carpinetana*, *Dritta*, *Gentile di Chieti* and *Intosso*) observing that two-phases decanter influenced the hydroxytirosol derivates and the quality and the nutraceutical properties of EVOO [37]. Regarding α-tocopherol, that increases EVOO stability in the presence of light, *Leccino*, *Intosso,* and *Rustica* cultivars exhibited the highest levels of this polyphenol [39]. Recently, through NMR spectroscopy, *Coratina* cultivar has been characterized in order to detect peculiar polyphenols [40]. This cultivar is the most popular EVOO variety of the Apulian region and it is qualified by bitterness and pungency. *Coratina* cultivar presents an elevated concentration of polyphenols (oleuropein and ligstroside) that are directly linked to the strong taste [40]. Furthermore, other Apulian *Picoline* and *Peranzana* cultivars exhibited a similar content of polyphenols [40].

These results confirm that olive products, and in particular EVOO, play a key role in health status and quality of life, thus confirming their importance as functional food with several potential clinical applications (Table 1). However, more studies, especially in humans, are needed to fully clarify the benefits of precise EVOO monocultivars.

## 3. Protective Role for EVOO on Human Health

Chronic diseases are the most prevalent conditions worldwide, with a high impact on public health. Although the incidence of chronic illness is exponentially growing, the associated prognosis is still limited, thus resulting in an increasing trend of mortality. Nowadays, it is estimated that more than 60% of all deaths are due to chronic illness (https://www.who.int/nutrition/topics/2_background/en/). Beside the genetic predisposition that usually characterize these diseases, a large amount of studies delineated how the environmental factors can together concur to determine the onset of chronic disease. Among them, diet probably represents one of the most important factors.

The consumption of EVOO rich in phenolic compounds has been linked to the promotion of antioxidant and anti-inflammatory responses, that collectively attenuate the progression of chronic illness [41]. The EFSA (European Food Safety Authority) has recently approved a claim that polyphenols protect against lipid peroxidation at a minimal dose of 5 mg/kg/day, equivalent to the consumption of 23 g of EVOO [42]. Specifically, the phenolic compounds bind to LDL particles and protect them against oxidation. High level of oxidized LDL in the plasma is considered a strong predictor of cardiovascular disease and has been widely associated with metabolic diseases, such as obesity, metabolic syndrome (MetS), and type 2 diabetes [43,44,45,46,47].

Even though more studies are needed, nowadays several evidences suggest that olive oil consumption improves risk factors for different diseases. In particular, replacing the saturated fatty acids typical of many dietary pattern, with the EVOO enriched in MUFA has been correlated with a reduced risk of cardiovascular disease and obesity, implying that the type of fat more than the quantity is important for achieving health benefits [48].

### 3.1. EVOO in Obesity, MetS, and Diabetes

The increased incidence of obesity and related metabolic alterations has become a growing pandemic emergency in the last years. The accumulation of visceral fat, a trademark of obesity, is usually the resultant of an imbalance between high energy food intake and sedentary lifestyle. Therefore, the adoption of a healthier lifestyle with an appropriate dietary pattern has generally been accepted as the main treatment for obese individuals as well as for people with MetS and type 2 diabetes [49]. In this context, the implementation of MD in the daily routine have proven to positively influence health outcomes. Particularly, supportive results indicated that the consumption of olive oil within the MD may prevent obesity and related metabolic diseases.

The EPIC-PANACEA prospective cohort study showed that the high adherence to the MD (including EVOO) was related to a decreased possibility to become overweight or obese [50]. A randomized dietary trial, the PREDIMED study, demonstrated that virgin olive oil intake for 3 years was associated with a limited body weight gain and a reduced waist circumference [51,52] Moreover, data from the Pizarra population-based cohort study, which analyzed 613 subjects over 6 years, showed a significant decrease in the obesity incidence in those who consumed olive oil than in those who consumed sunflower oil [53]. Although it could appear not easy to distinguish between the effects of MD and those of olive oil, since they are intrinsically related, several evidences support the beneficial consequences of olive oil per se in contrasting weight gain. The habitual use of olive oil enhances the palatability of salads, vegetables, and legumes, thus favoring the increase consumption of foods high in dietary fiber and low in energy density that promotes higher satiation and satiety [54]. Moreover, it has been described that the uptake of dietary oleic acid serves as a molecular sensor linking fat ingestion to satiety, by promoting the mobilization of intestinally-derived lipid messenger oleoylethanolamide [55]. Finally, MUFA contained in olive oil improve glucose homeostasis and increase diet-induced thermogenesis if compared to a diet rich in saturated fatty acids [56,57].

Increasing evidences are also indicating that olive oil could be a useful tool in the lifestyle management of MetS patients. Indeed, the SUN prospective study observed an inverse relationship between adherence to the MD and the cumulative incidence of the MetS [58]. However, not all the studies provide a precise correlation between the two, but underline that the implementation of olive oil MD in the habitual routine could ameliorate only individual aspect of MetS. The EPIC study showed that the adherence to MD lowers visceral fat accumulation, may be due to the effect of the high content of olive oil that prevents the redistribution of body fat from the peripheral to abdominal adipose tissue [59,60]. The PREDIMED trial demonstrated that MD intervention supplemented with olive oil results in the reduction of blood pressure and insulin resistance together with a downregulation of inflammatory biomarkers [61]. Finally, the LIPGENE study pointed out that the long term consumption of a MUFA rich diet attenuates the postprandial inflammatory state and serum lipidic profile associated with MetS [62,63].

More clear evidences have been found for the relation between olive oil and type 2 diabetes. First, the EPIC-InterAct project analyzed the adherence to the MD in 11,994 incident type 2 diabetic case subjects and a stratified sub-cohort of 15,798 participants, finding that people with high scores were less likely to develop type 2 diabetes compared to who had a lower score [64]. Next, studies confirmed that the protective effects were ascribable mostly to olive oil consumption. The strongest indication that olive oil may prevent type 2 diabetes comes from the PREDIMED study. This follow-up study that analyzed men and women without diabetes but at high cardiovascular risk found that participants following a MD enriched with EVOO presented a 40% reduced incidence of diabetes compared to control group [65]. Moreover, a multicentric parallel trial of the PREDIMED pointed out that MD supplemented with virgin olive oil decrease body weight and improves glucose metabolism, two important parameters closely related to the onset of type 2 diabetes [66]. Accordingly, a prospective cohort study on Spanish university graduates demonstrated that subjects with closed adherence to a MD had a lower risk of developing diabetes [67]. Finally, a recent metanalysis proved that the intake of olive oil could be beneficial for the prevention and the management of type 2 diabetes. However, these effects seems to be limited to the intake of olive oil as a whole, and not applicable to its single component [68].

### 3.2. EVOO in Cardiovascular Diseases

In the 1990s, the Lyon Diet Heart Study, a prevention trial testing the protective effects of a MD, indicated that increasing adherence to this type of diet reduced the risk of cardiovascular diseases [69]. Since then, a large plethora of studies were carried out in order to define the single food or nutrient entailed in cardio protection. This has allowed to widely recognized the fundamental role played by olive oil in the cardio-protective effect of MD. Indeed, olive oil (especially, virgin and extra virgin) has proved positive effects on many factors predisposing to cardiovascular diseases, including blood pressure, lipid profile, and endothelial function [41]. Specifically, the PREDIMED study reported that the consumption of EVOO resulted in a decrease risk of cardiovascular disease and mortality in individuals at high cardiovascular risk, owing to its capacity to improve lipid profile and decrease blood pressure which consequently lowers the risk of major cardiovascular events [61,70]. The EPIC-Spain prospective study associated a decreased risk of coronary heart disease and cardiovascular mortality to the consumption of total olive oil, highlighting that this association is strongest, with a greater decrease of coronary heart disease events, when virgin olive oil is consumed rather than the ordinary one [71,72]. The lower risk of mortality after myocardial infarction has been correlated to olive oil intake also in an Italian population, using data from the GISSI-Prevenzione clinical trial [73]. Moreover, a metanalysis described an inverse association between the consumption of olive oil and stroke events [74]. Finally, the PREDIMED study based on a multicenter trial in Spain on participants at high risk of cardiovascular disease who were randomly assigned to different dietary patterns, pointed out that MD supplemented with EVOO diminished the incidence of major cardiovascular events compared to low fat diet [75].

Recently, a systematic review and meta-analysis of cohort studies reported that a combination of MUFA, olive oil, and oleic acids is able to provide beneficial effects in terms of reduction of cardiovascular mortality, cardiovascular events, and stroke, although consumption of olive oil per se present a higher association with cardiovascular health [76]. A crossover study, the EUROLIVE, with the aim to evaluate the consequences of daily consumption of olive oil with different phenolic content, observed that high polyphenol olive oil as the EVOO improves plasma lipids levels and decreases oxidative damage, thus suggesting that this source of fat may provide additional benefits against cardiovascular risk factors [77]. Specifically, the protective effects of olive oil against cardiovascular and heart disease is mainly attributable to the high content of oleuropein and hydroxytyrosol, two phenolic compounds. These components have an antioxidant effect and lower platelet aggregation and monocyte adhesion while diminishing cardiotoxicity and coronary occlusion [78]. Even though these compounds are currently under investigation in both in vitro and in vivo studies [79], their potential as nutraceutical for the prevention of cardiovascular diseases is still to be determined.

## 4. Nutrigenomics of Different EVOO Cultivars

Nutrigenomics is defined as the study of food effects on gene expression. Starting from a deep dissection of the interaction between nutrients and the genome at molecular level, nutrigenomics shows the potential impact of a dietary regime or food constituents on human health [80]. Thanks to the impressive progress of omic technologies in the postgenomic era [81], the knowledge of EVOO functional properties in healthy or pathological conditions has grown considerably [82].

Here, we reported the principal human nutrigenomic studies that provide a clear relation between VOO and EVOO with different polyphenol content and their health impact. VOO and EVOO are both characterized by the same amount (high) of polyphenols, but differ for the free acidity grade that is greater in extra-virgin olive oil than in virgin olive oil. The studies taken into account were classified according to the following parameters; (1) dietary intervention: comparison of different VOO/EVOO polyphenol content alone or in the context of traditional Mediterranean Diet (TMD); (2) the administration timing of dietary intervention: postprandial or sustained consumption of VOO/EVOO; (3) the type of the study; (4) the sample population: healthy volunteers and/or patients; (5) the background diet; (6) the method used to assess dietary adherence; (7) the analyzed tissue; (8) the technical approach used for the gene expression analysis: microarray or real time-PCR (RT-PCR); (9) the target molecules studied: genes or miRNAs (Table 2). Peripheral blood mononuclear cells (PBMCs) are the most used target population because they act as carriers of systemic signals, thus the analysis of their transcriptome could be useful to identify new biomarkers of several diseases including inflammatory disorders ranging from MetS [83] to cancer [84] and/or to investigate the effect of dietary regimen administration [85]. Moreover, because of the critical role of PBMCs in the formation and repair processes of atherosclerotic lesions, they could potentially serve as diagnostic signature for atherosclerosis [86]. From a technical point of view, PBMCs offer the advantage to be easily available from the study population and isolated with a fast procedure.

Nutrigenomic studies describing a sustained consumption of VOO with a high polyphenol content (HPC) are able to modulate pathways related to inflammation, oxidative stress, and lipid metabolism as compared to olive oil containing low polyphenol content (LPC) in healthy population. In a subpopulation of the EUROLIVE study, RT-PCR data on PBMCs demonstrated that three weeks consumption of olive oil with high polyphenol content (366 mg/Kg) in healthy subjects reduced the activation of the CD40/CD40 ligand (CD40L) system and its downstream products as compared to olive oil with low polyphenol content (2.7 mg/Kg), and this reduction was associated with a decrease in plasma LDL oxidation [87]. Specifically, a down modulation of pro-inflammatory cytokines and the chemotaxis signaling pathways for monocytes and neutrophils are reported. Another paper on a subpopulation of the EUROLIVE study demonstrated the ability of *IL8RA* to modulate blood pressure because of its involvement in renin-angiotensin-aldosterone system (RAAS) regulation at the angiotensin II level [88]. Specifically, among the tested genes of RAAS system, *IL8RA* was the only one whose expression is significantly reduced in PBMCs isolated from healthy subjects after administration of 25 mL of EVOO rich in polyphenols when compared to the administration of olive oil with low polyphenol content for three weeks. Interestingly, the same pathways resulted modulated after a prolonged consumption of VOO. In fact, administration of EVOO with (328 mg/kg) or without (55 mg/kg, also termed “washed” olive oil, WOO) polyphenols for twelve weeks in the context of TMD, reduce the expression of genes related to inflammation (*IFNγ* and *IL-7R*), lipid metabolism (*ARHGAP15*), oxidative stress (*ADRB2*) and DNA repair (*POLK*) as compared to participants’ habitual diet. Even if these effects are induced by TMD *per se*, the authors demonstrated that these genes, except for *IL-7R* and *POLK*, followed a trend of reduction in TMD+WOO group that became statistically significant only when TMD dietary pattern was administered with high polyphenol content (TMD + VOO group) [89].

**Figure 1:**
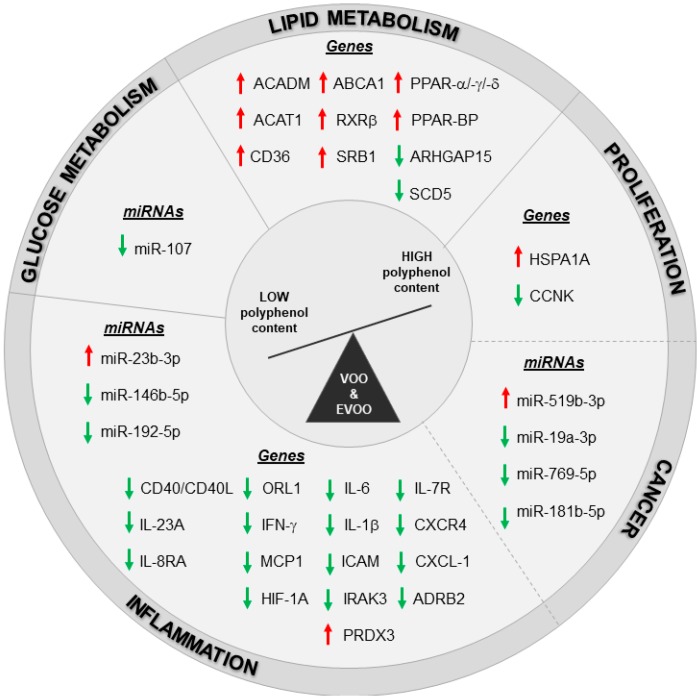
Transcriptomic changes induced by high polyphenols VOO and EVOO intake in healthy and unhealthy population. Red arrows: up-modulated genes/miRNAs, green arrows: down-modulated genes/miRNAs. Dashed line indicates an overlapping in the modulation of some genes and miRNAs between inflammation, proliferation and cancer pathways.

The pathways related to lipid metabolism and inflammation were regulated also with postprandial consumption of VOO and EVOO. Specifically, White Blood Cells (WBCs) isolated from prehypertension or stage 1 hypertension patients five hours after 30 mL intake of olive oil with a natural VOO with a medium polyphenol content (MPC, 289 mg/kg) and a high polyphenol content (961 mg/kg, obtained by the addition of a phenolic-rich extract from the olive cake to the MPC olive oil) were analyzed by RT-PCR [90]. Gene expression data revealed the promotion of cholesterol efflux process in WBC of patients with an acute intake of high- as compared to medium-polyphenol VOO content [90]. These pathways resulted modulated also in another study on PBMCs population reporting changes in genes and miRNAs expression of healthy subjects and MetS patients after an acute intake of different EVOO cultivars. Specifically, 50 mL of two Italian EVOO cultivars only differing for their polyphenol content were administered to both groups. These cultivars are the *Coratina* cultivars, enriched in polyphenols (491 mg/kg), and the *Peranzana* cultivar with a low polyphenol content (270 mg/kg) [32]. A paired analysis of microarray data (before and four hours after high-polyphenols EVOO administration), revealed the modulation of 2438 annotated genes (1376 up- and 1062 down-regulated genes) in PBMCs from healthy volunteers. The modulation of the cellular processes regulating lipid metabolism, proliferation, inflammation and cancer pathways was confirmed, with a partial overlapping among them (Figure 1). The modulation of healthy subjects’ transcriptome was in line with the positive effects observed on glucose metabolism by biochemical measurements following acute intake of polyphenol-enriched EVOO; these effects were not induced by low-polyphenol EVOO administration. A parallel microarray analysis on PBMCs from MetS patients showed a reduced number of genes (954 annotated genes: 403 up- and 551 down-regulated genes) significantly modulated by EVOO rich in polyphenols. The weaker changes in mRNA expression from MetS patients as compared to CTRL group reveals that the benefic effects of EVOO with high polyphenol content were partially lost in these patients. Importantly, candidate genes validated by RT-PCR did not show any significant changes in healthy subjects underwent acute intake of EVOO with low polyphenol content apart from *HSPA1A*, *RXRβ* and *CXCR4* that showed the same modulation induced by EVOO enriched in polyphenols. Therefore, these modulations can be ascribed to the EVOO mayor components (mono- and poly-unsaturated fatty acids) rather than to its minor components (polyphenols). The up-regulation of *RXRβ*, along with those of *RXRα*, in polyphenol-enriched EVOO parallels with the activation of *PPARα* and its coactivator PPARγ coactivator 1-alpha (*PGC-1α*) that was predicted by the pathway analysis and by IPA “upstream regulator” prediction tool. This trend was in line with the known interaction of retinoid X receptors and PPARs to form heterodimers [91,92] and with our previous findings reporting on PPARs and RXRα suppression in PBMCs from MetS patients [83]. Overall, data from this paper points to the importance of the phenolic fraction and of the health status of the subject receiving EVOO intake in order to achieve the most effective transcriptomic changes in PBMCs. Moreover, another postprandial study was reported on PBMCs isolated from MetS patients after the administration of two virgin olive oil-based breakfasts with high (398 ppm) and low (70 ppm) polyphenol content (the latter obtained by the physical extraction of most phenolic compounds from the high polyphenol content) [93]. Microarray data from the comparison of olive oil with high and low polyphenol content reported the modulation of proliferation, survival, and migration as cellular functions while the most represented network was the “inflammatory disorders.” The important role of the inflammatory response was verified in a subsequent paper by the same authors in an extended cohort of MetS patients [94]. The postprandial inhibition of pro-inflammatory gene expression induced by the breakfast with polyphenol-enriched VOO when compared to VOO-based breakfast with low polyphenol content was correlated with a lower plasmatic levels of lipopolysaccharides (LPS) and in turn to lower NF-kB activation, with a reduction in *IL-6*, *IL-1β*, and *CXCL-1* (C-X-C Motif Chemokine Ligand 1) expression in PBMCs from MetS patients [94].

In the context of the great advance in omic technologies, the study of non-coding RNAs shed a new light on transcriptomic studies, because of the ability of these molecules to control many biological processes. MicroRNAs (miRNAs) are an emerging class of noncoding RNAs able to regulate gene expression in tissues and biological fluids in the context of both physiological and pathological conditions like MetS, cardiovascular diseases, and cancer [83,95,96]. miRNAs are relatively stable single-stranded molecules, 19–23 nucleotide-long, which negatively modulate the expression of their target mRNAs at post-transcriptional level by binding to 3′-untranslated region of mRNAs [97]. miRNAs can also be secreted into the circulation by some type of cells (e.g., macrophages and platelets) and exert their regulatory effects on different cell populations by an endocrine or paracrine mechanism of action [98]. Circulating miRNAs could be considered as a promising non-invasive biomarkers to be used for diagnostic purposes [99]. For all these reasons, miRNAs could be very useful in nutritional science and could be used to test the pathways modulated by dietary treatments in healthy and/or unhealthy population [100]. To our knowledge, the paper by D’Amore et al. was the only one reporting the use of microarray technology applied to the miRNome profiling in PBMCs from healthy subjects and MetS patients after an acute intake of polyphenol-enriched EVOO [32]. The authors decided to focus on miRNome after the observation that argonaute RISC catalytic component 2 (*AGO2*), a gene involved in miRNAs processing, is down-modulated by EVOO rich in polyphenols in PBMCs from healthy volunteers [101], thus explaining why most of the miRNAs validated by RT-PCR were suppressed. Specifically, miRNAs involved in inflammation (miR-146b-5p [102], miR-181b-5p [103], and miR-192-5p [104]), cancer (miR-19a-3p [105], miR-181b-5p [103], and miR-769-5p [106]), and disorders linked to glucose metabolism (miR-107 [107]) were down-modulated after a single dose of EVOO rich in polyphenols. The only two miRNAs that were up-regulated were involved in anti-inflammatory and tumor suppressing processes (miR-23b-3p and miR-519b-3p, respectively) [108,109,110]. As demonstrated in the same paper by data from gene expression, miRNAs changes were not confirmed in MetS patients receiving EVOO with high polyphenol content (except for the down modulation of miR-19a-3p); this could be explained by the absence of modulation in *AGO2* gene even if more studies are needed to validate this hypothesis. On the contrary, the miRNome profiling of PBMCs from an extended population of MetS patients at basal level showed a high number of modulated miRNAs mainly involved in the regulation of innate and adaptive immune responses as compared to healthy subjects [111]. Specifically, in our recent work we identified miR-9-5p as a direct regulator of ABCA1 in circulating CD14^+^ cells in patients with MetS thus revealing a new mechanism for cholesterol efflux regulation able to reduce the cardio-metabolic risk [111].

All the nutrigenomic studies described here have demonstrated the ability of polyphenol-enriched EVOO to act on transcriptome and miRNome, promoting human health thanks to its anti-inflammatory, anti-cancer and anti-oxidant properties, and to its modulation of glucose/lipid metabolism (Figure 1). 

**Table 2:** Transcriptomic studies describing the impact of VOO and EVOO characterized by different polyphenol content on human population.

Dietary Intervention	Administration Timing	Type of the Study	Study Population	Background Diet	Method Used to Identify Dietary Pattern	Tissue	Technical Approach	Target Molecule	Ref.
HPC vs. MPC VOO	Post prandial (acute intake)	Randomized, double-blind, crossover, controlled trial	Pre/hypertensive patients from Spain	2-week washout period (during the week before the intervention: 10% of saturated fatty acids; on the day before: polyphenol-free diet.	3-day dietary record	WBCs	RT-PCR	Genes	[90]
HPC vs. LPC EVOO	Post prandial (acute intake)	Paired study	Healthy subjects and MetS patients from Italy	1-week washout period (no olive oil); 3 days before the intervention: low-phenolic compound diet.	-	PBMCs	Microarray	Genes/miRNAs	[32]
HPC vs. LPC VOO	Post prandial (VOO-based breakfast)	Randomized, double-blind, crossover trial	MetS patients from Spain	6-week washout period (low fat, CHO diet, no vitamins and soy supplements); on the day before the intervention: no phenol-rich food.	3-day dietary record and FFQ	PBMCs	Microarray	Genes	[93]
HPC vs. LPC VOO	Post prandial (VOO-based breakfast)	Randomized, crossover trial	MetS patients from Spain	6-week washout period (low fat, CHO diet, no vitamins and soy supplements); on the day before the intervention: no phenol-rich food.	3-day dietary record and FFQ	PBMCs	RT-PCR	Genes	[94]
HPC vs. LPC VOO	Sustained consumption (3 weeks)	Randomized, crossover, controlled trial	Healthy subjects from Finland, Germany, and Spain (subgroup of EUROLIVE study)	2-week washout period (no olives and olive oil)	3-day dietary record	PBMCs	RT-PCR	Genes	[87]
HPC vs. LPC VOO	Sustained consumption (3 weeks)	Randomized, double-blind, crossover trial	Healthy subjects from Finland, Germany, and Spain (subgroup of EUROLIVE study)	2-week washout period (no olives and olive oil)	3-day dietary record	PBMCs	RT-PCR	Genes	[88]
TMD+VOO TMD+WOO Control diet	Sustained consumption (12 weeks)	Randomized, parallel, controlled trial	Healthy subjects from Spain	-	FFQ	PBMCs	RT-PCR	Genes	[89]

HPC: high polyphenol content, MPC: medium polyphenol content, LPC: low polyphenol content, VOO: virgin olive oil, EVOO: extra virgin olive oil, TMD: traditional Mediterranean diet, MetS: metabolic syndrome, WBCs: white blood cells, PBMCs: peripheral blood mononuclear cells, CHO: carbohydrate rich diet.

A common limitation for the reported studies is the sample size that does not take into account the high interindividual variability. In the case of studies with a sustained VOO and EVOO consumption, another limitation is the potential interaction between the compound tested in the study and other dietary components that might affect the results. Moreover, more studies are needed to verify if the beneficial effects of polyphenols-enriched VOO/EVOO will be retained after a prolonged administration timing and to understand if these effects are derived from one phenolic compound or are promoted by a synergistic effect of the total phenolic fraction.

## 5. Conclusions

Nutrigenomic studies reveal that EVOO cultivars characterized by a high polyphenol content are able to modulate the expression of several transcripts and miRNAs involved in different pathways, i.e., glucose/lipid metabolism, proliferation, inflammation, and cancer supporting health-promoting effects. For this reason, polyphenols have to be considered as an active and important player rather than a minor component of EVOO in the context of nutrigenomic modulation. Thus, the positive impact of EVOO on human health could be ascribed to a synergic effect of polyphenol compounds with the high content of oleic acid.

The introduction of high-throughput techniques is very helpful for the study of nutrigenomics induced by EVOO and could be used to discover nutritional biomarkers for prevention and/or prognosis of human diseases.

Apart from the studies summarized in this review regarding EVOO effects on transcriptomics, some other papers using omic technologies to analyze the modulation of the proteome and the metabolome induced by EVOO are reported [112,113]. To date, the integration of data generated by omic technologies is still missing. In the nearest future, a holistic strategy combining genomic, proteomic, and metabolomic data would be essential to understand the biological meaning of the results obtained at cell, tissue, and organ level in term of health benefits derived from the different fatty acids and polyphenol composition of EVOO cultivars.

## Figures and Tables

**Table 1:** Extra-virgin olive oil (EVOO) and olive oil clinical trials.

Trial Identifier	Trial Phase (Status)	Disease	Intervention
Obesity			
NCT03101436	Completed	Obesity	Dietary supplement: extra virgin olive oil and red wine
NCT03024359	Recruiting	Obesity	Dietary supplement: extra virgin olive oil
NCT03441802	Completed	Obesity	High quality extra virgin olive oil
NCT02463435	Completed	Severe obesity	Behavioral: nutritional intervention Nutritional intervention plus olive oil Dietary supplement: olive oil
Diabetes and Hypertension			
NCT03891927	Not yet recruiting	Insulin resistance	Dietary supplement: extra virgin olive oil
NCT03447301	Not yet recruiting	Type 2 diabetes mellitus	Dietary supplement: extra virgin olive oil (30 mL daily)
NCT02831803	Completed	Hypertension	Dietary supplement: walnuts Dietary supplement: extra virgin olive oil
Cardiovascular diseases			
NCT03528603	Recruiting	Platelet aggregation Nutritional and metabolic disease Cardiovascular diseases	Oleocanthal-rich extra virgin olive oil Oleocanthal-low extra virgin olive oil
NCT03053843	Recruiting	Atrial fibrillation Atrial arrhythmia	Dietary supplement: Mediterranean diet plus extra virgin olive oil
NCT03796780	Recruiting	Cardiovascular risk factor	Dietary supplement: extra-virgin olive oil Dietary supplement: refined olive oil
NCT03105947	Completed	Cardiovascular risk factor	Coconut oil Butter Olive oil
NCT03683134	Completed	Cardiovascular Diseases Cardiovascular risk factor Obesity	Behavioral: Mediterranean diet Dietary supplement: olive oil and mixed nuts Behavioral: American Heart Association
NCT03005535	Unknown	Atherosclerosis	Vitaminized corn oil Olive oil
